# Cefotaxime-associated encephalopathy in peritoneal dialysis patients: A case report

**DOI:** 10.1097/MD.0000000000042915

**Published:** 2025-06-20

**Authors:** Gao Song, Rong Li, Na Yang, Meng-Qun Cheng, Cai-Qiong Zhang

**Affiliations:** a Department of Pharmacy, Puer People’s Hospital, Pu’er, China; b Department of Pharmacy, Ning’er County Hospital of Traditional Chinese Medicine, Pu’er, China; c Department of Reproductive Medicine, Puer People’s Hospital, Pu’er, China.

**Keywords:** CAE, case report, cefotaxime, delirium, metabolic encephalopathy

## Abstract

**Rationale:**

Cefotaxime is a widely used 3rd-generation cephalosporin antibiotic that is primarily excreted through urine. Third-generation cephalosporins (e.g., ceftazidime, ceftriaxone) and the 4th-generation cefepime are associated with neurological complications. Although cephalosporin-associated encephalopathy (CAE) is well-documented, cases specifically induced by cefotaxime remain remarkably rare. To our knowledge, this is the 1st documented case of cefotaxime-induced CAE in a patient undergoing peritoneal dialysis.

**Patient concerns:**

A 72-year-old female patient on peritoneal dialysis developed delirium and impaired consciousness 7 days after receiving cefotaxime as part of her anti-infective therapy for a pulmonary infection. The patient expressed concern regarding the onset of impaired consciousness and intermittent delirium as well as her prognosis and potential sequelae.

**Diagnoses:**

This investigation focused on the potential role of cefotaxime in triggering CAE in peritoneal dialysis patients. The likelihood of cefotaxime causing drug reactions was confirmed using the Naranjo Scale score.

**Interventions:**

Following the suspicion of cefotaxime-induced encephalopathy, the treatment regimen was modified, which included discontinuation of cefotaxime, initiation of intravenous piperacillin/bactam as an alternative antibiotic therapy, and symptomatic treatment with olanzapine. Close monitoring and adjustments to the peritoneal dialysis regimen are essential for managing neurological symptoms.

**Outcomes:**

After discontinuation of cefotaxime and appropriate adjustments to the treatment regimen, the patient’s clinical condition gradually improved. Symptoms of delirium subsided, consciousness returned to normal, and laboratory indicators stabilized.

**Lessons:**

This case emphasizes the importance of considering the potential adverse effects of antibiotics, particularly in patients with kidney insufficiency undergoing dialysis. This highlights the necessity of vigilance in monitoring drug-related complications for timely detection of encephalopathic symptoms and development of individualized treatment strategies. We acknowledge that cefotaxime may increase the risk of CAE in patients on peritoneal dialysis, necessitating heightened awareness among physicians.

## 1. Introduction

Cefotaxime is a 3rd-generation cephalosporin antibiotic typically administered 2–3 times/d. cefotaxime is predominantly eliminated via renal excretion (approximately 50% of the administered dose recovered in urine as unchanged drug), with a smaller proportion excreted via biliary pathways (<10%).^[1,2]^ Compared to 40% clearance by continuous kidney replacement therapy,^[3]^ peritoneal dialysis removes only 5% of cefotaxime.^[4]^ Delirium is a common and costly neurological complication associated with hospitalization,^[5]^ and both acute and chronic kidney insufficiency are significant risk factors for adverse neurological effects.^[6]^ Patients with kidney insufficiency are particularly susceptible to cephalosporin-associated encephalopathy (CAE); however, existing literature has predominantly focused on ceftazidime, cefepime, and ceftriaxone.^[7–9]^ There are few reports concerning cefotaxime, with only 1 English-language case report from 1990 documenting CAE due to high-dose cefotaxime in a patient undergoing hemodialysis,^[10]^ as well as 2 non-English-language reports,^[11,12]^ for which detailed information remains unavailable. In this report, we describe the case of a patient with kidney disease who developed impaired consciousness and intermittent delirium despite receiving an appropriate dose of cefotaxime for 7 days. Our findings indicate that even when cefotaxime is administered to patients undergoing peritoneal dialysis and the dose is adjusted based on blood creatinine levels, CAEs may still occur, warranting continued vigilance from healthcare providers.

## 2. Case presentation

**Figure 1.**
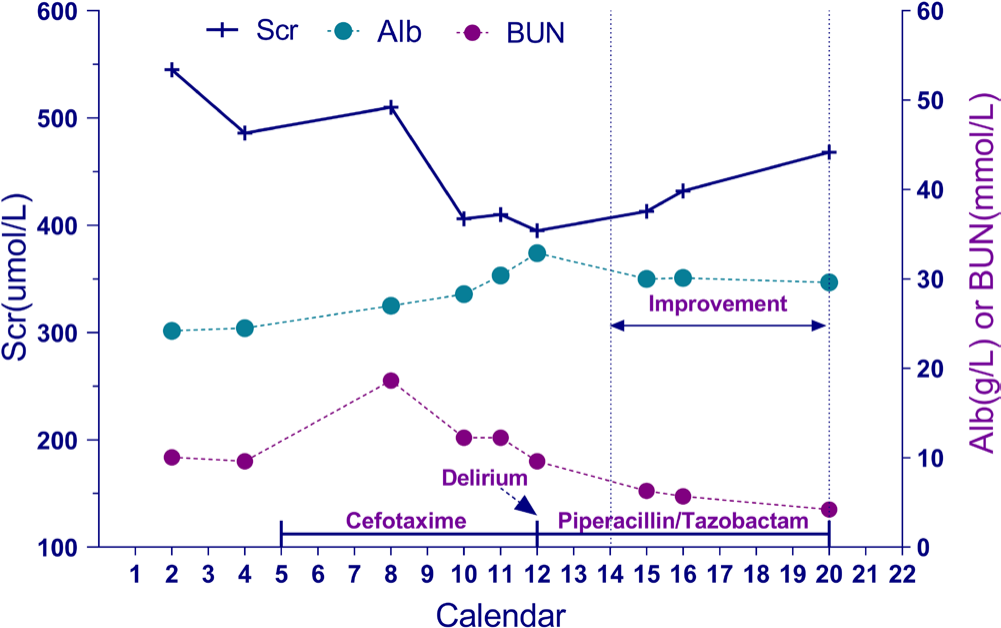
Patient’s hospitalization and treatment timeline. The figure illustrates the patient’s hospitalization course, including treatment interventions and laboratory parameters. Alb = albumin, BUN = blood urea nitrogen, Scr = serum creatinine.

A 72-year-old female patient (height, 150 cm; weight, 38 kg) with stage 5 chronic kidney disease was on long-term peritoneal dialysis. She was admitted to the hospital after exacerbation 8 hours earlier due to clumsiness of movement and weakness of the extremities for 3 years. The patient’s medical history included microcephaly for 3 years and congenital heart disease. On day 5 of admission, the patient developed a fever and cough. Chest computed tomography (CT) showed bilateral pulmonary infiltrative solid lesions and multiple inflammatory nodules with mild elevation of the calcitonin gene (0.30 ng/mL), and pulmonary infection was considered (Table S1, Supplemental Digital Content, https://links.lww.com/MD/P213). Upon reviewing the patient’s medical history, it was found that 4 months prior, during an episode of hospitalization, G+ cocci – staphylococci was cultured from both the sputum and ascitic fluid. The infection was successfully treated with intraperitoneal administration of cefotaxime and cefazolin, after which the patient resumed regular dialysis. Given that no antibiotics had been administered since the treatment, and the last infection episode occurred more than 3 months ago, the risk of antimicrobial resistance was considered low intermediate. Therefore, for the initial anti-infection treatment regimen, we considered cefotaxime 1 g q24h iv.gtt for intravenous therapy (Fig. 1).

Due to poor treatment of the pulmonary infection, the patient developed impaired consciousness after 2 days of adjusted treatment with intravenous cefotaxime (1 g, q12h, iv.gtt), which manifested in a lethargic state, intermittent delirium, and involuntary tremors in both upper extremities. During the episode of delirium, the Glasgow Coma Scale score was 12 (eye response: 3, verbal response: 4, motor response: 5). Cranial magnetic resonance imaging (MRI) on admission showed mild acute-phase infarction and lacunar softening foci, but a subsequent review of cranial CT did not reveal any significant abnormalities (Figs. S1–S3, Supplemental Digital Content, https://links.lww.com/MD/P213). After a multidisciplinary consultation, we concluded that this was metabolic encephalopathy (nephroencephalopathy) and suspected that cefotaxime antibiotics might have been an important factor contributed to the development of the patient’s encephalopathy. Therefore, the cefotaxime injection was discontinued and adjusted to the patient’s infectious condition, treatment was switched to intravenous piperacilatazobactam (4.5 g, q12h, iv.gtt), and olanzapine was administered for symptomatic treatment and an adjusted peritoneal dialysis regimen (Table S2, Supplemental Digital Content, https://links.lww.com/MD/P213). After discontinuing cefotaxime and adjusting the regimen, the patient’s consciousness gradually increased, his psychiatric symptoms improved significantly, he no longer experienced delirium, and his urea nitrogen level was normal.

The medications used daily during hospitalization included atorvastatin calcium, esomeprazole, metoprolol, potassium chloride, ferrous succinate, wake-up calls, and human albumin (see Table S2, Supplemental Digital Content, https://links.lww.com/MD/P213 for details). We assessed cefotaxime-induced adverse drug reactions using the Naranjo Scale with a score of 6, and the relevance of the results was probable (Table 1).

**Table 1:** The Naranjo Scale for assessing causality.

Question	Yes	No	sDon’t know	Score	Reasons for scoring
1. Are there previous conclusive reports on this reaction?	+1	0	0	1	Encephalopathy associated with cefotaxime drugs has been reported
2. Did the adverse event appear after the suspected drug was administered?	+2	−1	0	2	Impaired consciousness and intermittent delirium following cefotaxime administration
3. Did the adverse event improve when the drug was discontinued or a specific antagonist was administered?	+1	0	0	1	After discontinuation of the drug, the patient gradually regained consciousness without delirium, that is, the adverse drug reaction was reduced.
4. Did the adverse event reappear when the drug was readministered?	+2	−1	0	−1	Not reused
5. Are there alternative causes that could on their own have caused the reaction?	−1	+2	0	2	Cefotaxime can be considered as an independent factor in causing encephalopathy in a patient with renal failure in this case
6. Did the reaction reappear when a placebo was given?	−1	+1	0	0	not applicable
7. Was the drug detected in blood or other fluids in concentrations known to be toxic?	+1	0	0	0	No determination of whether cefotaxime reached toxic concentrations
8. Was the reaction more severe when the dose was increased or less severe when the dose was decreased?	+1	0	0	0	The reaction did not resolve with dose reduction of cefotaxime
9. Did the patient have a similar reaction to the same or similar drugs in any previous exposure?	+1	0	0	0	Hospitalized 6 months ago with a combination of ceftazidime and cefazolin without this reaction
10. Was the adverse event confirmed by any objective evidence?	+1	0	0	1	Observed by specialists and documented in cases
Total score: 6					**/**

A total score of ≥9 indicates a definite causal relationship between the drug and adverse reactions, supported by objective evidence and quantifiable data. A total score of 5 to 8 suggests a probable association, with objective evidence or quantitative test results supporting the link. A total score of 1 to 4 suggests a possible association, indicating a situation that cannot be fully confirmed or completely ruled out. A total score ≤ 0 is considered suspicious, suggesting a coincidental or essentially unrelated occurrence (https://www.ncbi.nlm.nih.gov/books/NBK548069).

## 3. Discussion

Cefotaxime is a β-lactam antibiotic widely used to treat various infectious diseases. However, studies have indicated that β-lactam antibiotics may induce neurotoxicity,^[6]^ which can manifest as vigilance disorders, confusion, myoclonus, localized signs, hallucinations, and other symptoms. Beta-lactams account for nearly 50% of the adverse reactions to antibiotics reported by the French Drug Safety Warning System, with over 60% of these cases classified as serious adverse reactions.^[13]^ The issue of drug-induced neurological adverse reactions has not received sufficient attention within the medical community, despite being a significant contributor to increased morbidity and mortality among patients. However, the relationship between antibiotics and delirium remains unclear. Nevertheless, a recent retrospective study involving 100 critically ill patients identified a correlation between the use of 4th-generation cephalosporins, cefepime, and the onset of encephalopathy, with an incidence rate of up to 15%.^[14]^ Delirium is associated with prolonged hospitalization, heightened in-hospital comorbidities, and increased mortality within 1 year.^[15–17]^ Thus, timely recognition and prevention of delirium can enhance patient prognosis and reduce healthcare expenditures.^[18]^ For patients with diminished kidney function, particularly those who are chronically dependent on dialysis, increased attention must be directed towards the safety of antibiotic use. As kidney function deteriorates, the ability of the kidneys to eliminate drugs diminishes, resulting in drug accumulation in blood and tissues, which increases the risk of drug-induced adverse reactions. As cefotaxime is primarily eliminated through the kidneys, the potential for its accumulation is greater in patients with kidney insufficiency, thereby increasing the risk of cefotaxime-related delirium.

The patient was an elderly woman with stage 5 chronic kidney disease. We analyzed 3 potential causes of delirium symptoms. First, the patient’s blood pressure remained stable during the episode of delirium, and there were no abnormalities in blood glucose, electrolytes, or signs of metabolic acidosis, effectively ruling out hypotension, hypoglycemia, or electrolyte disorders as contributors to neurological symptoms. Second, cranial magnetic resonance imaging performed upon admission revealed mild acute-phase infarction and lacunar softening foci; however, a subsequent review of cranial CT did not show any significant abnormalities, thereby excluding organic encephalopathy. Nonetheless, cerebral infarction may have contributed to the development of adverse neurological reactions.^[6]^ Finally, we considered the possibility of adverse drug reactions. We hypothesized that delirium symptoms could be associated with the use of cefotaxime, as no other medications administered to the patient were identified as potential neurotoxic agents. Cefotaxime can induce central nervous system effects through several mechanisms: prolonged serum half-life in patients with reduced kidney function, resulting in increased blood concentrations; enhanced blood-brain barrier permeability in uremia, which may lead to an imbalance in central nervous system neurotransmitters and trigger neurological symptoms^[19]^; and decreased binding of cefotaxime (with protein binding of 30–50%) to albumin. Hypoproteinemia can elevate the free concentration of drugs in the bloodstream, and our patient had a serum albumin concentration below 30 g/L before the onset of encephalopathy. The Naranjo Scale score supports our hypothesis. It is noteworthy that, in addition to cephalosporin antibiotics, non-cephalosporin antibiotics such as penicillin, quinolones, and aminoglycosides have also been reported to induce similar encephalopathies in patients with uremia, although the mechanism of action remains unclear and may be related to cerebral GABA receptors.^[5,6]^ Research data indicate that 3rd-generation cephalosporins may cause neurological adverse effects, with an onset occurring between 1 and 10 days.^[20]^ These effects can manifest in individuals aged 1 to 91 years.^[21]^ Improvement is typically observed within 24 hours to 1 month following the discontinuation of the antibiotics, which is consistent with our case.^[22]^ However, it is essential to recognize that the efficacy of kidney replacement therapy varies based on the specific drug and the dialysis method employed. Hemodialysis is ineffective in removing ceftriaxone and cefoperazone, yet it is effective in eliminating cefotaxime.^[23]^ Conversely, peritoneal dialysis is ineffective in removing cefotaxime due to its lower permeability.

Our study suggests that, in some patients, such as those on peritoneal dialysis, the use of this drug may result in serious central adverse effects, although the dose was adjusted according to kidney function. Unfortunately, we were unable to obtain appropriate data to support this, as the patients refused to undergo cerebrospinal fluid blood concentration measurements. More high-quality evidence is needed as a next step to support drug dosage selection in this group of patients for more individualized drug therapy.

## 4. Conclusion

This case underscores the necessity of considering the potential adverse effects of antibiotics, particularly in patients with kidney insufficiency undergoing dialysis. This emphasizes the importance of vigilance in monitoring drug-related complications to facilitate the timely detection of encephalopathic symptoms and the implementation of individualized treatment strategies. We recognize that cefotaxime may increase the risk of CAE in patients on peritoneal dialysis, necessitating physicians to maintain a heightened level of awareness.

## Author contributions

**Conceptualization:** Gao Song, Cai-Qiong Zhang.

**Data curation:** Gao Song, Rong Li, Na Yang, Meng-Qun Cheng, Cai-Qiong Zhang.

**Formal analysis:** Gao Song, Rong Li, Na Yang, Meng-Qun Cheng, Cai-Qiong Zhang.

**Funding acquisition:** Gao Song.

**Investigation:** Meng-Qun Cheng.

**Methodology:** Gao Song, Rong Li, Meng-Qun Cheng, Cai-Qiong Zhang.

**Project administration:** Gao Song.

**Resources:** Meng-Qun Cheng.

**Software:** Gao Song, Meng-Qun Cheng.

**Supervision:** Cai-Qiong Zhang

**Validation:** Rong Li, Na Yang.

**Writing – original draft:** Gao Song, Rong Li.

**Writing – review & editing:** Gao Song, Meng-Qun Cheng, Cai-Qiong Zhang.

## Supplementary Material


