# Supplementary material for: Cefotaxime-associated encephalopathy in peritoneal dialysis patients: A case report
Source: Medicine (Baltimore). 2025 Jun 20;104(25):e42915. doi: 10.1097/MD.0000000000042915 (PMC12187338; doi:10.1097/MD.0000000000042915)
Supplement: Supplementary file 1 [file medi-104-e42915-s001.doc]

Supplemental tables

Table S1 Laboratory findings of the patient.

|  | Day 2 | Day 4 | Day 8 | Day 10 | Day 11 | Day 12 | Day 13 | Day 15 | Day 16 | Day 20 |
| --- | --- | --- | --- | --- | --- | --- | --- | --- | --- | --- |
| WBC(×10^9^/L) | 6.73 | 5.33 | 5.95 | 4.90 | 4.62 | NR | 6.27 | 5.76 | 4.77 | 4.72 |
| N(%) | 80.1 | 74.6 | 71.6 | 72.4 | 72.1 | NR | 74.9 | 79.7 | 68.3 | 69.2 |
| Plt(×10^9^/L) | 134 | 156 | 163 | 154 | 120 | NR | 148 | 149 | 156 | 139 |
| Na^+^(mmol/L) | 136 | 139 | 142 | 140 | 137 | 140 | 140 | 139 | 140 | 139 |
| K^+^(mmol/L) | 2.86 | 3.20 | 4.95 | 3.89 | 4.72 | 3.87 | 3.26 | 3.83 | 3.59 | 2.64 |
| Ca2^+^(mmol/L) | 2.21 | 2.25 | 2.26 | 2.21 | 2.04 | 2.02 | 1.92 | 1.96 | 1.89 | 1.96 |
| Alb(g/L) | 24.2 | 24.5 | 27.0 | 28.3 | 30.4 | 32.9 | NR | 30.0 | 30.1 | 29.6 |
| CRP(mg/L) | NR | 74.74 | NR | NR | NR | NR | 9.94 | 8.01 | 9.97 | NR |
| Pct(ng/ml) | NR | 0.30 | 0.32 | 1.32 | NR | 1.11 | NR | NR | 0.66 | 0.37 |
| BUN(mmol/L) | 10.05 | 9.60 | 18.63 | 12.23 | 12.25 | 9.59 | NR | 6.30 | 5.68 | 4.2 |
| Scr(umol/L) | 545 | 486 | 510 | 406 | 410 | 395 | NR | 413 | 432 | 468 |
| GLU(mmol/L) | 8.42 | 12.63 | 8.50 | 9.49 | 12.12 | 6.22 | NR | 8.41 | 5.99 | NR |
| GFR(mL/min) | 4.93 | 5.53 | 5.27 | 6.62 | 6.55 | 6.80 | NR | 6.51 | 6.22 | 5.74 |
| AST(U/L) | 16 | 34 | 49 | 19 | 19 | 22 | NR | 14 | 12 | 14 |
| ALT(U/L) | 14 | 29 | 36 | 32 | 15 | 18 | NR | 15 | 12 | 16 |
| ALP(U/L) | 144 | 151 | 113 | 92 | 61 | 78 | NR | 74 | 62 | 63 |
| γ-GTP(U/L) | 66 | 74 | 69 | 64 | 36 | 48 | NR | 54 | 48 | 62 |
| TC(mmol/L) | 3.99 | NR | 3.59 | NR | NR | NR | NR | NR | NR | NR |
| TG(mmol/L) | 1.91 | NR | 1.12 | NR | NR | NR | NR | NR | NR | NR |
| LDL(mmol/L) | 1.82 | NR | 1.95 | NR | NR | NR | NR | NR | NR | NR |
| T-Bil(umol/L) | 8.2 | 6.0 | 8.1 | 7.5 | 10.9 | 15.5 | NR | 11.7 | 9.5 | 8.3 |
| D-Bil(umol/L) | 4.2 | 3.4 | 3.9 | 3.7 | 5.1 | 6.4 | NR | 5.9 | 4.9 | 5.1 |

Note. WBC, white blood cell count; N, percent neutrophils; Plt, platelet count; Alb, albumin; CRP, C-reactive protein; BUN, urea nitrogen; Scr, serum creatinine; GFR, glomerular filtration rate (Cockcroft-gault); AST, aspartate aminotransferase; ALT, alanine aminotransferase; ALP, alkaline phosphatase; γ-GTP, gamma-glutamyl transpeptidase; T-Bil, total bilirubin; D-Bil, direct bilirubin; NR, no data.

Table S2 Major therapeutic drugs

| drugs | Dosage and Administration | Period(Days) |
| --- | --- | --- |
| Atorvastatin Calcium Tablets | 20mg qn po | D1-D18 |
| Ginkgo Biloba Extract Injection | 20ml qd iv.gtt | D1-D9 |
| Potassium Chloride Injection | 7ml qd iv.gtt | D1-D9 |
| Potassium Chloride Extended Release Tablets | 1g tid po | D12 |
| Cefotaxime for Injection | 1g qd iv.gtt/1g q12h iv.gtt | D6-D10/D11-D12 |
| Esomeprazole for Injection | 40mg bid iv.gtt  40mg qd iv.gtt | D11-D13  D14-D18 |
| Metoprolol Tartrate | 12.5mg bid iv.gtt | D11-D18 |
| Sobriety Injection | 20mg qd iv.gtt | D11-D18 |
| 1.5% Peritoneal Dialysis Solution | 2000ml tid Peritoneal Dialysis  2000ml bid Peritoneal Dialysis | D1-D12  D13-D20 |
| 2.5% Peritoneal Dialysis Solution | 2000ml bid Peritoneal Dialysis  2000ml tid Peritoneal Dialysis | D1-D12  D13-D20 |
| Ferrous Succinate Tablets | 0.2g bid po | D13-D18 |
| Piperacillin sodium tazobactam for injection | 4.5g q12h iv.gtt | D13-D20 |
| Human Albumin (20%) | 10g qd iv.gtt  20g bid iv.gtt  10g qd iv.gtt | D3-D9  D10,D13,D15  D16-D18 |
| Olanzapine Tablets | 5mg qn po | D11-D20 |
| Human Erythropoietin Injection | 6000IU st ih | D12 |

Nota: qd, once daily; qn, once daily at night; bid, twice daily; tid, three times daily; po, Oral; iv.gtt, intravenous; ih, subcutaneous injection

Supplemental figure

Supplemental Figure 1 On day 2 of hospitalization the patient underwent a cranial MRI


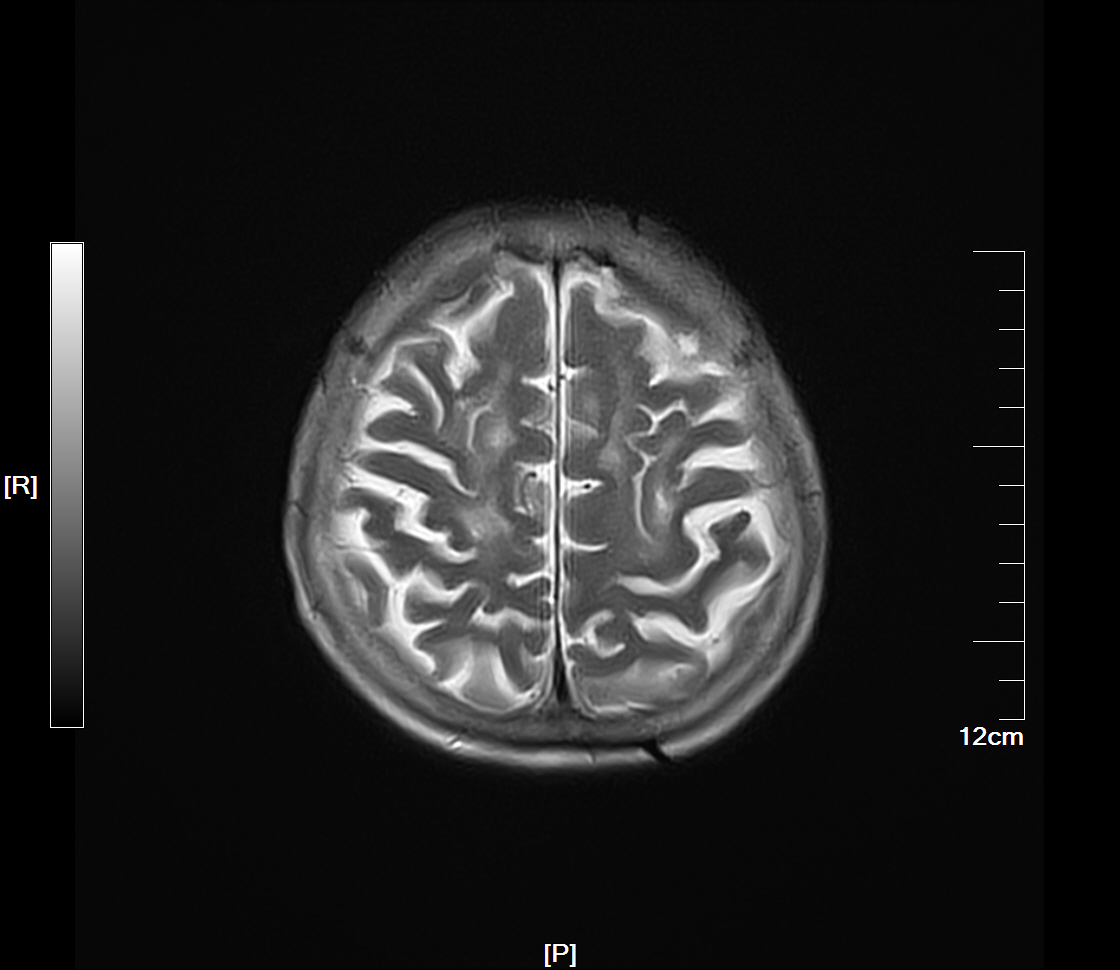

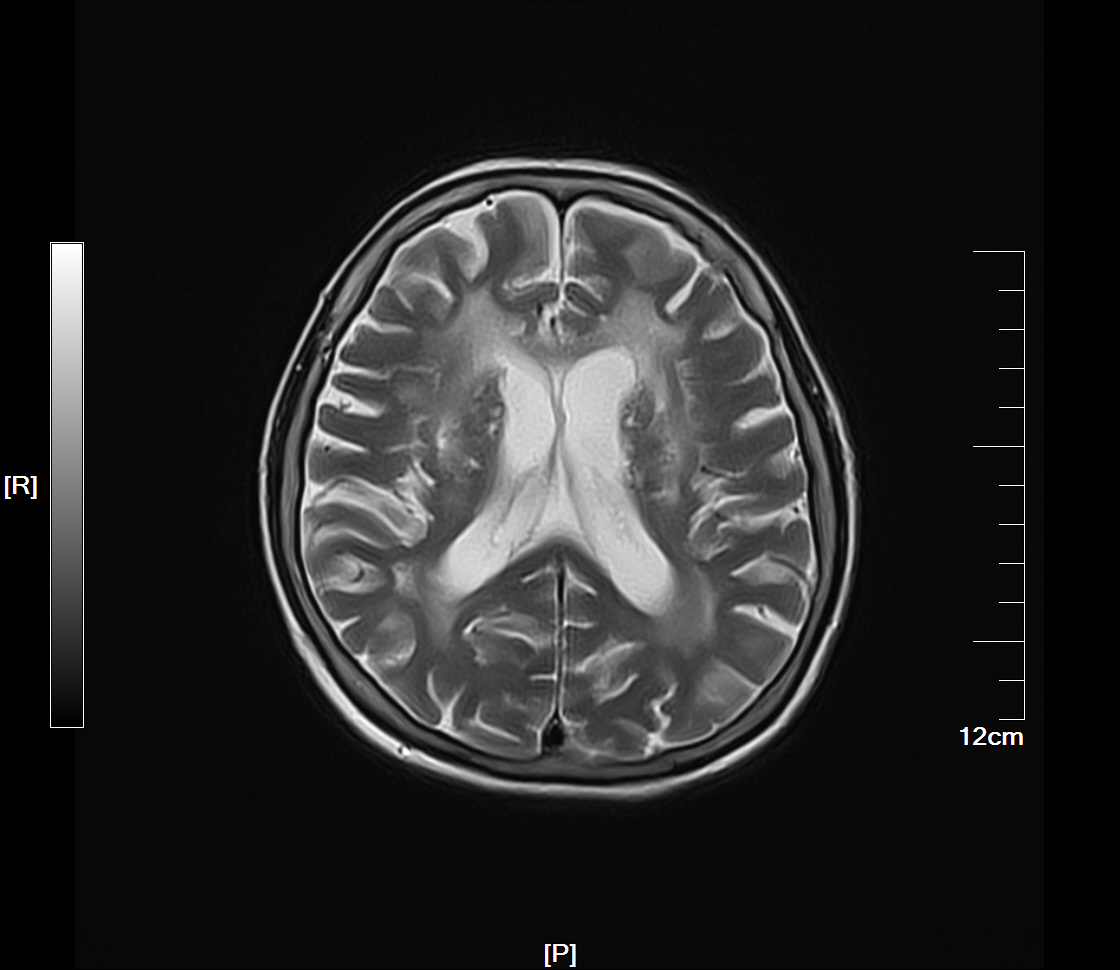


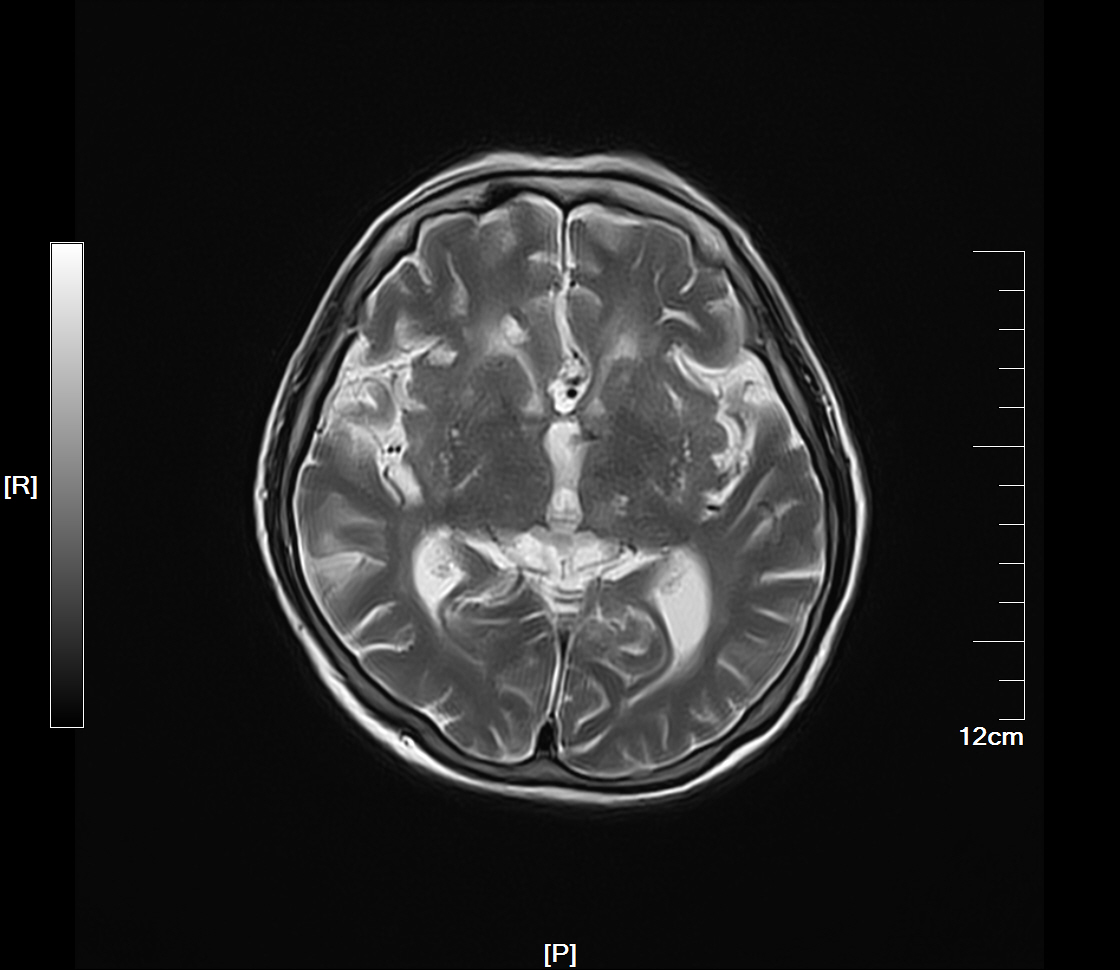
m
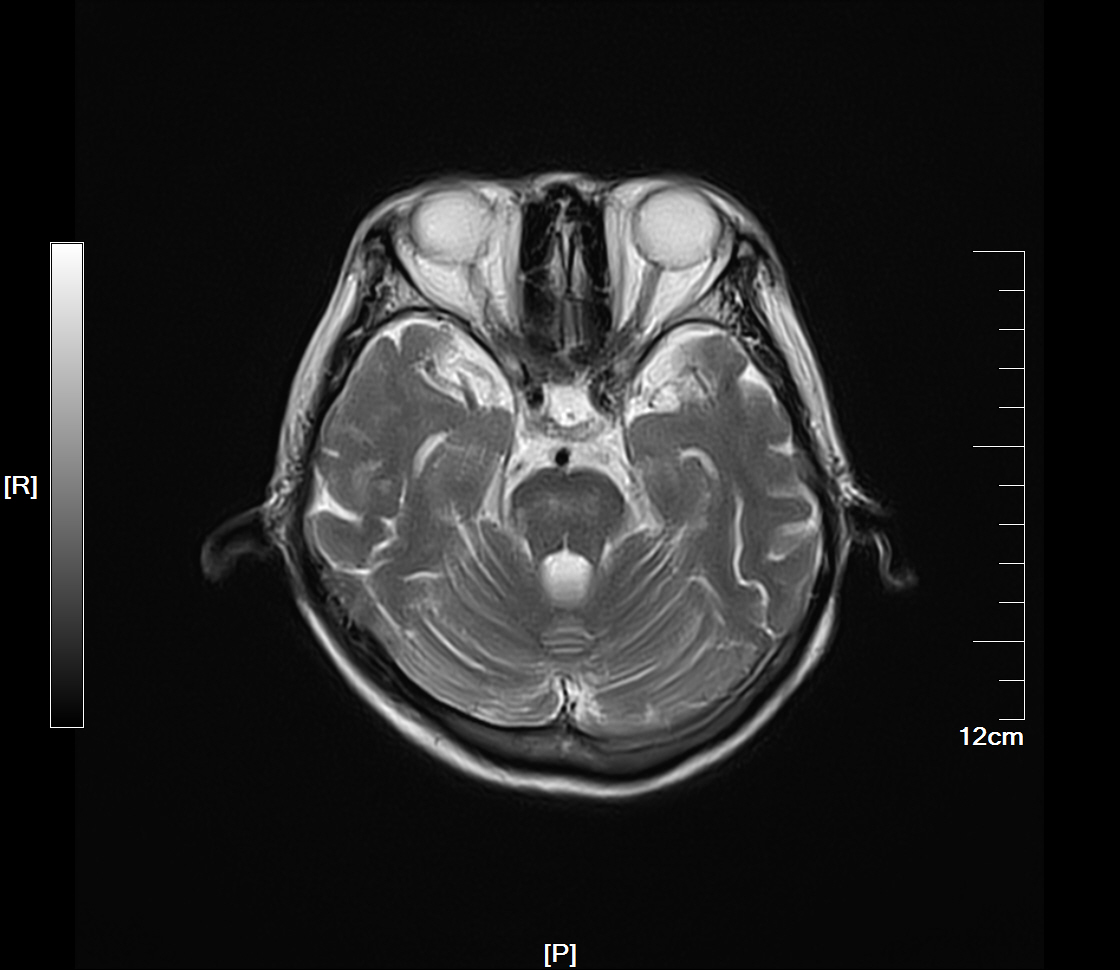


Reported results:1. few foci of acute-phase infarction in the right radial crown, parietal lobe and corpus callosum.

2. multiple old lacunar infarct foci and lacunar softening foci in the cerebral bridges, bilateral basal ganglia regions and radiocorona.

3. scattered microhemorrhagic foci in the pons, bilateral cerebellar hemispheres, basal ganglia region and cerebral hemispheres. Consider cerebrovascular amyloidosis.

Supplemental Figure 2 On day 13 of hospitalization the patient underwent a cranial computed tomography


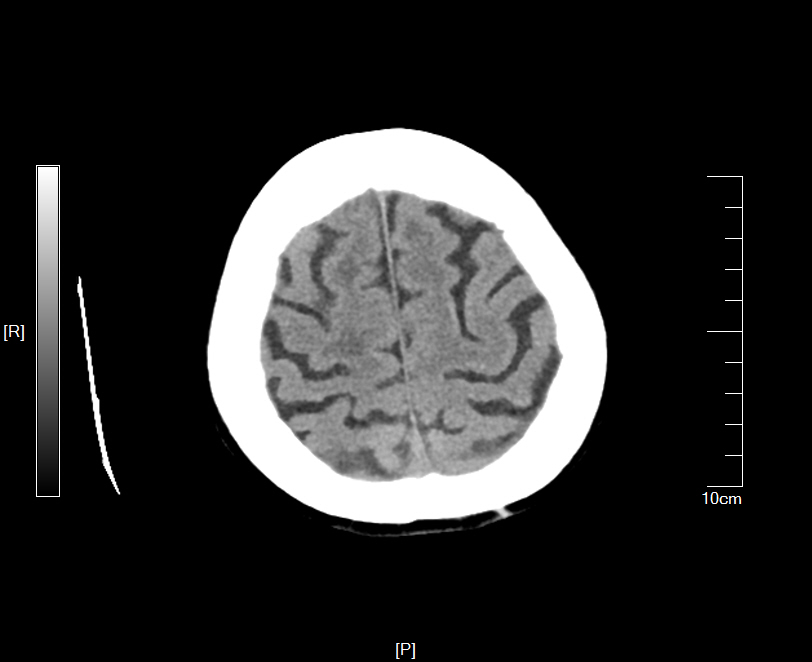

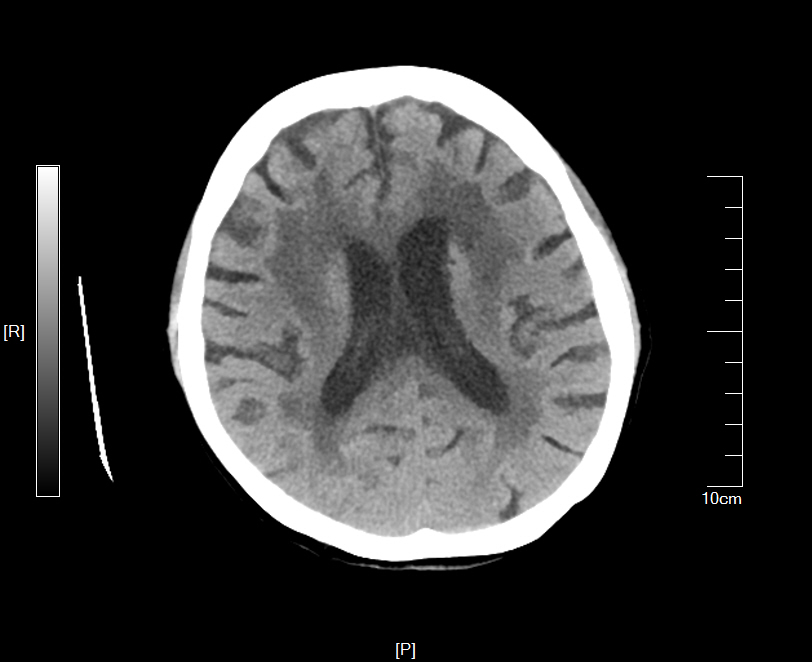


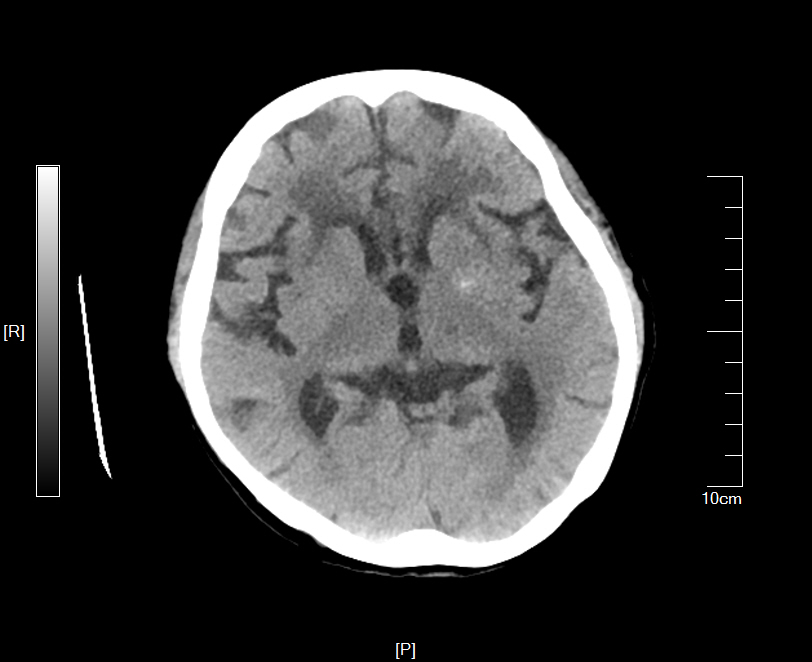

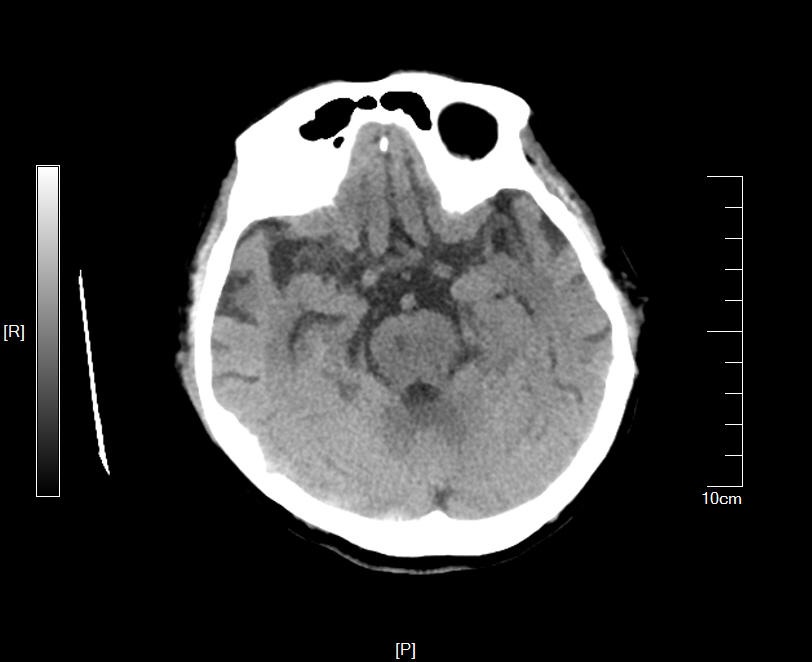


Reported results:1. Multiple lacunar foci in the pons, bilateral basal ganglia area and radial crown.

1. Bilateral frontoparietal lobe and periventricular white matter hypodensity in the lateral ventricles, please combine with MRI.

Supplemental Figure 3 On day 17 of hospitalization the patient underwent a cranial computed tomography


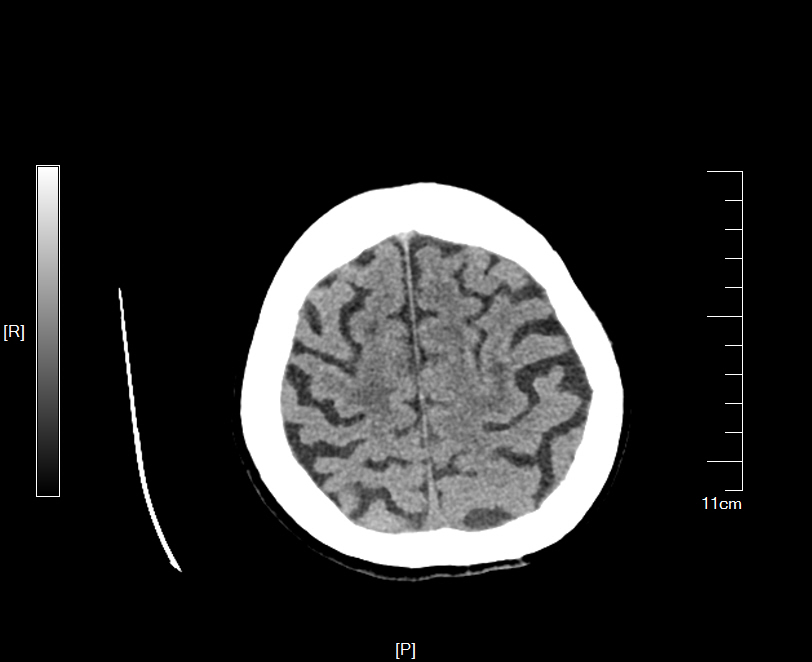

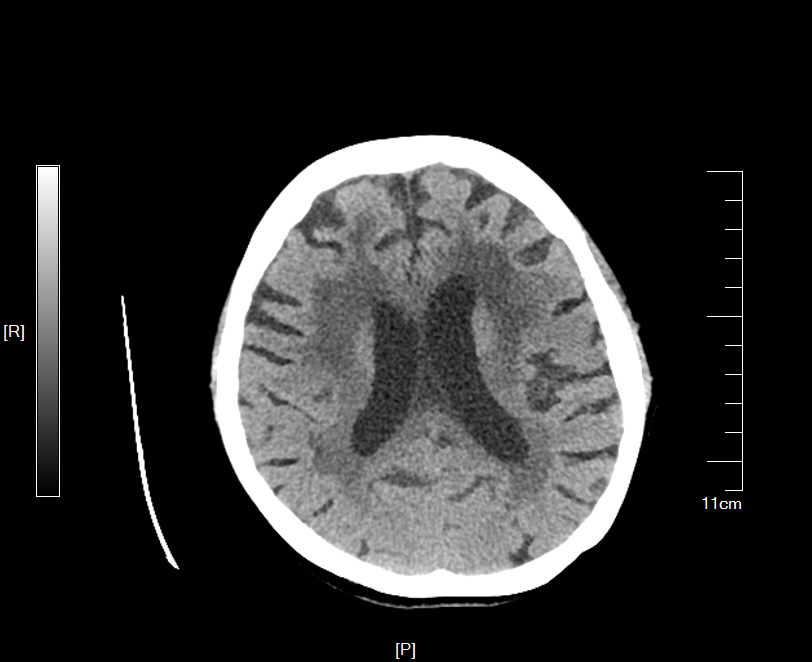


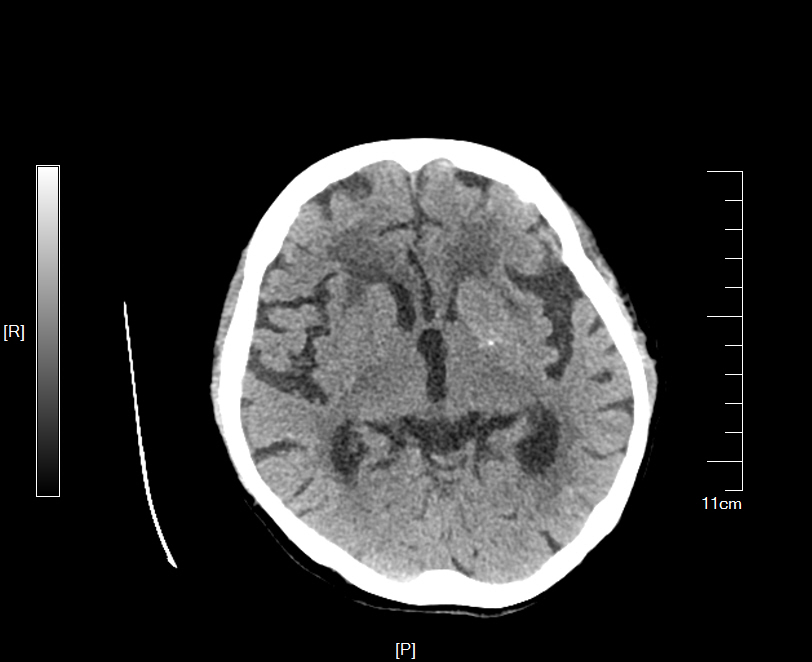

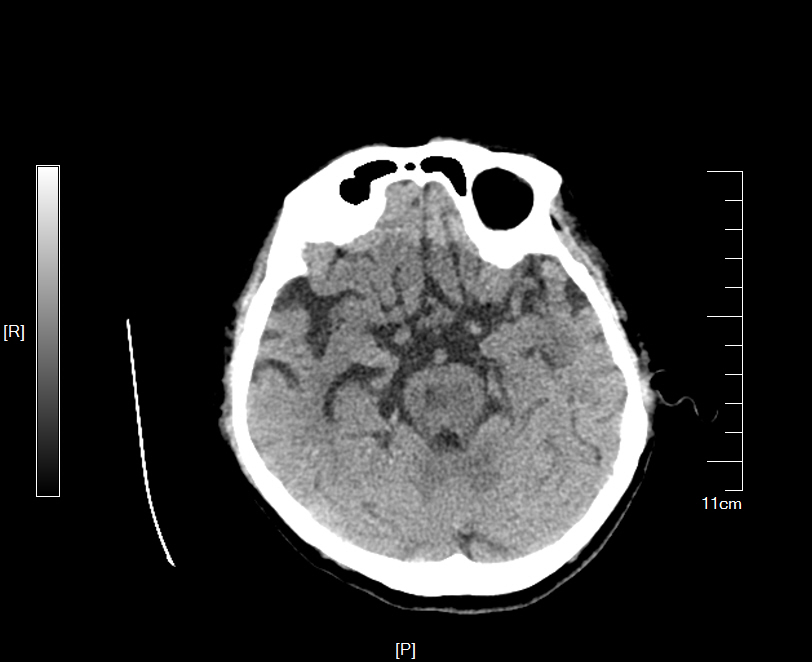


Reported results:Same as 9.27 report results
